# Supplementary material for: No impact of performance-based financing on the availability of essential medicines in Burkina Faso: A mixed-methods study
Source: PLOS Glob Public Health. 2022 Mar 23;2(3):e0000212. doi: 10.1371/journal.pgph.0000212 (PMC10021144; doi:10.1371/journal.pgph.0000212)
Supplement: Attachment — Submitted filename: Manuscript-PBF-BF-Drugs_responses-reviewers.docx [file pgph.0000212.s002.docx]

**Responses to reviewers**

Reviewer #1

| **Comment** | **Response** |
| --- | --- |
| Research question and relevance: A priori, I don't think there is any reason to think that a convoluted purchasing mechanism such as PBF could or should have any impact on the availability of essential medicines at the health facility level, especially in the absence of any specific interventions targeting essential medicine availability (and especially when facility autonomy is not well implemented). Essential medicines only constitute 2 percent of the quality checklist and even then it's a dummy yes/no variable. While the paper is well-done, I'm not exactly sure why a paper specifically on essential medicine availability - which seems to have been only tangentially incorporated as a policy objective of PBF - is relevant. It is clear that PBF is not very effective for improving the availability of essential inputs which should already be a given in any health system - there is no ex ante reason to reward health facilities to possess basic inputs which they should already have. It is unclear as to what percentage of stockouts were due to poor ordering or lack of medicines at the national warehouses to begin with. All of this to say that these caveats should come across more clearly. | Thank you for pointing out that we needed to make the – so far fairly implicit – ToC clearer, specifically why we did have reason to expect a change in the EM supply situation, and why a specific paper on medicines is of value.  In this context, we realized that we had forgotten to include the fact that drug availability was further incentivized in the quality checklist through correct application of treatment protocols for basic health services. Here, elements requiring drug availability contributed with about 12% to the overall quality score.  We have made the following changes to the manuscript:   - Description of the medicine supply situation prior to PBF to underline gaps in availability and room for change, which could have been impacted by PBF (p. 5, line 12 ff) - Inclusion of an explicit Theory of Change, where we highlight the role not only of the direct incentives, but also of indirect incentives and financial and supply chain management more generally (p. 8, line 5 ff) |
| Timing of quantitative analyses and linking changes in essential medicines availability to other indicators incentivized by PBF: It would be interesting to show a time dimension on the change of the indicator on essential medicines, as well as whether the change in this indicator was higher or lower compared to the change in other indicators, both in terms of the quality index as well as any quantity indicators. This would help contextualize the essential medicines indicator further in the context of the PBF program. It would be relevant to show what happened to other indicators in the same period: the paper alludes to quantity indicators improving faster given the incentives, many of which could only improve if essential medicines and commodities at the facilities are available; what is the reason behind this disconnect? Maybe it's just a question of when the indicators on essential medicines were collected, which is less continuous than quantity indicators? This could help clarify the disconnect between the quantitative and qualitative results. | In relation to the first point raised in this comment, we agree with the reviewer that it would be interesting to conduct an analysis to examine the timing of development of different indicators, to capture whether there is a relationship between changes in one indicator (in this case availability of EM) and changes in another indicator (for example, service provision). Unfortunately, however, data at our disposal do not allow for such an analysis. As described in the manuscript, we derived quantitative data for this analysis exclusively from two surveys, one conducted prior to the PBF launch and one conducted three years into the program. This means that we do not observe the availability of EM at different points in time, but exclusively at baseline and at endline. This is the case for all indicators we monitored in the study. Hence, we are not in the position to examine changes over time nor the relationship between changes across indicators quantitatively. We address how EM availability has probably fluctuated over the course of the intervention in the qualitative findings.  In relation to the second point raised in this comment, i.e. the perceived disconnection between quantity and quality indicators, we did not intend to refer to an observed disconnect (measured by our analysis), but rather to information we collected qualitatively, with providers explaining how they paid more attention to incentives related to quantity than to quality targets, as those paid more. It is our assumption that this may be one of the reasons explaining why the program did not produce considerable changes in the availability of EM. |
| Focus more on context, change pathways and theory of change: The paper could benefit from a stronger conceptual framework, which has been put forth in a similar evaluation of the Cameroon program (Sieleunou et al 2019) as well as another paper from Cote d'Ivoire (Duran et al 2020) which also used a qualitative approach. It would be good to unpack the specific pathways through which the program in Burkina Faso evaluated in this program could have changed essential medicine availability. This would also help crispen the recommendations on how the program or other health system interventions could be better designed; focusing on facility autonomy, role of regional authorities/strengthened decentralized governance, as well as the integration of diverse purchasing programs (e.g. getting rid of the gratuite program). This should also proactively indicate what is in the decision space of facilities versus not. The discussion or conclusion sections should more proactively indicate implications of these results, both for the future of the PBF program (is this still ongoing? Integrated to a national strategic purchasing program? Why or why not?) | Many thanks again for pointing out that the ToC needs to be clearer. We have changed the structure of the introduction by adding sub-headings, we have added background information on the drug supply situation pre-PBF, and we have spelled out the assumed theory of change in response to your suggestion, modelled after the Cameroon paper.  We have also added some detail on PBF’s current status to the discussion (p. 29, line 9 ff). With the government firmly committed to the gratuité for a number of reasons, discussions of retaining the quality component and/or integrating it into the gratuité have been on-going since 2018, with no success yet, in part due to the issues with measurement of quality discussed in the paper.  Finally, we have expanded the conclusions slightly, but purposely held back in providing more in terms of policy recommendations as – as you rightfully pointed out in your first comment – the paper provides a glimpse into only one aspect of PBF. |

Reviewer #2

| **Comments** | **Responses** |
| --- | --- |
| The introduction would benefit from a clear explanation of the mechanism by which the authors expect PBF to decease stock-outs or increase EM supply (perhaps around lines 66-68). This hypothesized mechanism appears to be referenced in the results section later in the paper (lines 318-321). If this is indeed the researchers’ hypothesis, it should not be included in the results section but in the introduction as suggested. It could then be referred to in the results as to whether the qualitative research disproved or proved their hypothesis. | Thank you for pointing out that the paper would benefit from an explicit formulation of the Theory of Change. We have added this starting from page 8, line 5 ff. |
| The qualitative data collection section in the methods could be improved by adding some additional detail, specifically, which participants IDIs were conducted with as opposed to FGDs, and why the different methods were chosen per group. The description of the qualitative sample is very long and hard to follow – it might be more impactful to present in table format. Were any other data collected on the qualitative participants other than role (i.e., age, gender, location, etc.?) | We have transformed the description of the qualitative sample into a table as suggested, and added that focus groups were only performed with communities. FGDs for this group were chosen in the context of the overall qualitative study, which at community level had focused on community dynamics around a community-based targeting of the ultra-poor which was also part of PBF, but irrelevant in the context of medicines.  We did collect additional data on respondents, but do not believe that they have any relevance to the findings, and therefore do not present them so as to keep the paper focused. |
| While there is a lot of valuable information in the qualitative results section, in its current format it is so long and full of detail that the reader becomes lost. I have two primary suggestions to help with this:  3a. The quotes used, while valuable in that they highlight the voices of the respondents, are overly long. I recommend reviewing the quotes and trimming text to make them more succinct while staying true to the respondents' views; others could be summarized in a sentence or two rather than quoted. A nice rule of thumb may be 1 direct quote per theme. Additional quotes could also be presented in a table.  3b. Again, due to the length of the results section it becomes difficult for the reader to keep all of the qualitative findings in their head at once. A figure summarizing the qualitative themes and how they interact might be a nice component to add. | Thank you for pointing out that the quotes interrupt rather than support flow. As we feel that they are important nonetheless, we followed the suggestion to move them to a table (Table 4), and also trimmed them where possible without losing meaning.  We have further summarized the findings graphically, as suggested, in relation to the Theory of Change, see new Figure 1 on p. 25 |
| I suggest changing the title by excluding “underlining the importance of context and intervention design.” It is an interesting discussion point but does not come across as the main takeaway of the article (rather what comes across is that PBF did not increase EM availability). | We have done as suggested |
| Is a medicine really considered available if there is only 1 unit present? What if 2 patients are present that day and need the same medicine? Are there standard definitions of medication availability? It would be helpful to either have a citation for the definition of medication availability or brief explanation from the authors regarding this choice. | This indicator definition was chosen in alignment with the PBF indicator definition. However, we are aware and concur with its inherent limitation, particularly in the context of PBF, and have added a sentence to the limitations section accordingly. |
| Perhaps personal preference, but I suggest using “purposive” or “purposively” sampled rather than “purposely.” | We have changed this in line with the suggestion. |
| I suggest removing the respondent reference IDs from quotes – they do not add anything for the reader. | We have changed this in line with the suggestion. |
